# Effectiveness and safety of finerenone in diabetic kidney disease patients: a real-world observational study from China

**DOI:** 10.1080/0886022X.2024.2400541

**Published:** 2024-09-09

**Authors:** Jingying Zhou, Le Kang, Chenjie Gu, Xinwei Li, Xianan Guo, Ming Fang

**Affiliations:** a Department of Nephrology, The First Affiliated Hospital of Dalian Medical University, Renal Translational Medicine Center of Liaoning Province, Dalian, China; b Medical College of Dalian University, Dalian, China

**Keywords:** Finerenone, diabetic kidney disease, albuminuria, hyperkalemia

## Abstract

**Aims:**

Finerenone has been approved for treating diabetic kidney disease (DKD) with reducing cardiorenal risk. Real-world data on finerenone treatment for the management of DKD are presently lacking. This study aimed to investigate the effect of finerenone on the renal parameters of the Chinese DKD population in the real-world medical setting for the first time, especially in combination with renin–angiotensin system inhibitors (RASi) and sodium-glucose cotransporter 2 inhibitors (SGLT2i).

**Methods:**

Forty-two DKD patients were selected and completed a 6-month finerenone treatment. Renal parameters and adverse effects were collected at every visit.

**Results:**

The median urine albumin-to-creatinine ratio (UACR) was 1426.11 (755.42, 3638.23) mg/g. Among them, the proportion of patients with a UACR of 300–5000 mg/g was 76.2%, and the proportion of patients with a UACR of >5000 mg/g was 14.3%. The median estimated glomerular filtration rate (eGFR) was 54.50 (34.16, 81.73) mL/min/1.73 m^2^. Finerenone decreased the UACR significantly throughout the study period (*p* < .05). The maximal decline of UACR at month 6 was 73%. Moreover, the proportion of patients with a 30% or greater reduction in UACR was 68.42% in month 6. There was a smaller decline (9–11%) in the eGFR after initiating finerenone (*p* > .05). One patient each discontinued finerenone due to hyperkalemia (2.4%) and acute kidney injury (2.4%). No patient reported hypotension, breast pain, and gynecomastia.

**Conclusions:**

This study from China first demonstrated finerenone decreased UACR with manageable safety in real-world DKD treatment. A triple regimen of RASi, SGLT2i, and finerenone may be a promising treatment strategy for lowering albuminuria and reducing hyperkalemia risk in advanced DKD patients.

## Introduction

Diabetic kidney disease (DKD), which leads to chronic kidney disease (CKD) and end-stage kidney disease (ESKD), is a major public health problem globally [1–3]. During the past decades, understanding of the pathophysiology of DKD and treatment of DKD has progressed. In addition to controlling hyperglycemia, hypertension, and hyperlipidemia, pharmaceutical drugs like renin–angiotensin system inhibitors (RASi) and sodium-glucose cotransporter 2 inhibitors (SGLT2i) are mainly used in treating DKD. However, the residual risk of cardiorenal adverse outcomes persists with these guideline-directed medical therapies despite addressing renal hyperfiltration and metabolic disturbances [4–6]. Evidence from animal and human studies confirmed that perturbations in pro-inflammatory and pro-fibrotic pathways due to the overactivation of mineralocorticoid receptors (MRs) play a key role in the development of cardiorenal disease [7].

Finerenone, a selective nonsteroidal MR antagonist (MRA), has been approved in many countries including the US, Europe, Japan, and China. In several recent landmark randomized clinical trials, finerenone reduced the cardiorenal risk of type 2 diabetes and CKD with manageable adverse events (AEs) such as hyperkalemia [8–10]. Finerenone has been evidently incorporated into the guideline-directed medical treatment for albuminuria in DKD [11,12]. However, hyperkalemia was frequently seen (14%) with finerenone treatment when compared to placebo [9,13], although the risk of discontinuation due to hyperkalemia is low. Real-world data on finerenone treatment for the management of DKD are presently lacking, particularly regarding the efficacy of finerenone in combination with RASi and SGLT2i in China. This study aimed to investigate the safety and renal effect of finerenone in patients with DKD in routine clinical practice.

## Methods

### Study design and setting

This single-center, retrospective cohort study was conducted from December 2022 to December 2023 at the Nephrology Section of the First Affiliated Hospital of Dalian Medical University. All participates gave their informed consent and personal identity information was anonymized. This study complied with the Declaration of Helsinki and was approved by the local hospital ethics committee (approval number PJ-KS-KY-2024-84).

### Patient selection and inclusion criteria

**Figure 1.**
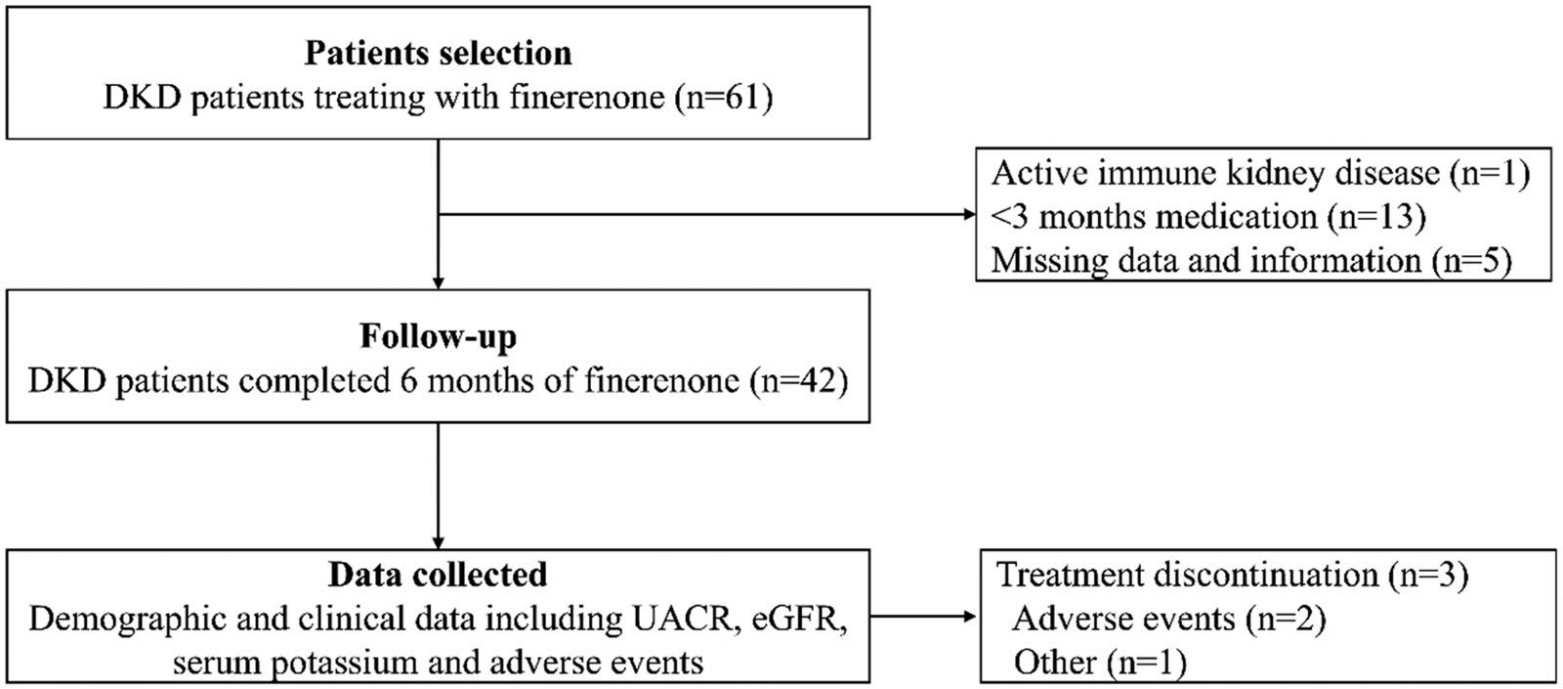
The flowchart of patient selection and treatment. DKD: diabetic kidney disease; UACR: urine albumin-to-creatinine ratio; eGFR: estimated glomerular filtration rate.

Adult CKD patients (≥18 years old) with urine albumin-to-creatinine ratio (UACR) ≥30 mg/g and estimated glomerular filtration rate (eGFR) ≥25 mL/min per 1.73 m^2^ were screened. Inclusion criteria were: (1) patients diagnosed with DKD based on medical history and clinical presumption, or a pathological diagnosis suggesting diabetic nephropathy; (2) patients treated with finerenone according to the drug instruction; (3) patients with a follow-up period of no less than 6 months. Exclusion criteria were: (1) patients with concurrent active immune kidney disease, autosomal dominant polycystic kidney disease, or other causes of kidney disease; (2) patients treated with finerenone for no more than 3 months; (3) patients with incomplete follow-up data or who were lost to follow-up. The flowchart of patient selection and treatment is shown in Figure 1.

### Data collection

Demographic and clinical characteristics were collected at baseline. After initiating finerenone treatment, all patients were followed up at months 1, 3, and 6, and every 1–3 months thereafter. Urine albumin-to-creatinine ratio, serum potassium, and eGFR were gathered for the effectiveness and safety analyses of finerenone. Adverse events were also recorded and updated on the follow-up times.

### Statistical analysis

Statistical analysis was performed using SPSS version 26.0 (SPSS, Chicago, IL) and R (4.2.0), along with Zstats version 0.90 (www.medsta.cn/software). The primary and safety data analyses were performed on the full analysis set. Normally distributed data were compared by fitting an analysis-of-covariance model to the data at 1, 3, and 6 months to those of baseline while nonnormally distributed data were subjected to logarithmic transformation or generalized estimation equations. The least squares mean of the ratio of UACR, eGFR, and serum potassium values at 1, 3, and 6 months to those of baseline were calculated in covariance analysis. The analysis results were given by point estimation (least squares mean) and a 95% confidence interval.

A *post hoc* analysis examined the proportion of patients with a UACR reduction of at least 30%, 30–50%, and at least 50% from baseline at each visit.

## Results

### Baseline data

Forty-two DKD patients were included in the analysis for the study period. The patient demographic and clinical characteristics at baseline are shown in Table 1. The mean age of the participants was 61.02 ± 13.55 years; among them, 66.7% were males and 92.9% of participants had cardiovascular disease. At baseline, 85.7% of the patients received RAS inhibitors, 90.5% of the patients received SGLT2 inhibitors, and 14.3% received glucagon-like peptide-1 (GLP-1) receptor agonists. The median UACR was 1426.11 (IQR: 755.42, 3638.23) mg/g. The proportion of UACR of 300–5000 mg/g was 76.2%, and the proportion of UACR of >5000 mg/g was 14.3%. The median eGFR was 54.50 (IQR: 35.04, 83.73) mL/min/1.73 m^2^. The mean serum potassium was 4.55 ± 0.55 mmol/L (Supplementary Table 1).

**Table 1.** Patient baseline characteristics.

Characteristics	Total (*n* = 42)
Age, mean ± SD, years	61.02 ± 13.55
Male sex, *n* (%)	28 (66.7%)
Hypertension history, *n* (%)	39 (92.9)
Duration of diabetes (≥10 years), *n* (%)	28 (66.7)
History of cardiovascular disease, *n* (%)	39 (92.9)
Serum potassium, mean ± SD, mmol/L	4.55 ± 0.55
eGFR, median (IQR), mL/min/1.73 m^2^	54.50 (35.04–83.73)
≥60 mL/min/1.73 m^2^, *n* (%)	11 (26.2)
45–60 mL/min/1.73 m^2^, *n* (%)	27 (64.3)
25–45 mL/min/1.73 m^2^, *n* (%)	4 (9.5)
UACR, median (IQR), mg/g	1426.11 (755.42–3638.23)
UACR, *n* (%)	
30–300 mg/g	4 (9.5)
300–3000 mg/g	27 (64.3)
3000–5000 mg/g	5 (11.9)
≥5000 mg/g	6 (14.3)
Baseline medications, *n* (%)	
Renin–angiotensin system inhibitors	36 (85.7)
β-Blockers	10 (23.8)
Calcium channel blockers	25 (59.5)
Diuretics	11 (26.2)
Calcium polystyrene sulfonate	1 (2.4)
Statins	33 (78.6)
Glucose-lowering medications	
Insulin and analogues	19 (45.2)
Metformin	8 (19.0)
Sulfonylureas	1 (2.4)
α-Glucosidase inhibitors	7 (16.7)
DPP-4 inhibitors	3 (7.1)
SGLT-2i	38 (90.5)
GLP-1 receptor agonists	6 (14.3)

SD: standard deviation; eGFR: estimated glomerular filtration rate; IQR: interquartile range; UACR: urine albumin-to-creatinine ratio; DPP-4: dipeptidyl petidases-4; SGLT2i: sodium-glucose cotransporter 2 inhibitor; GLP-1: glucagon-like peptide-1.

### Effectiveness analysis

**Figure 2.**
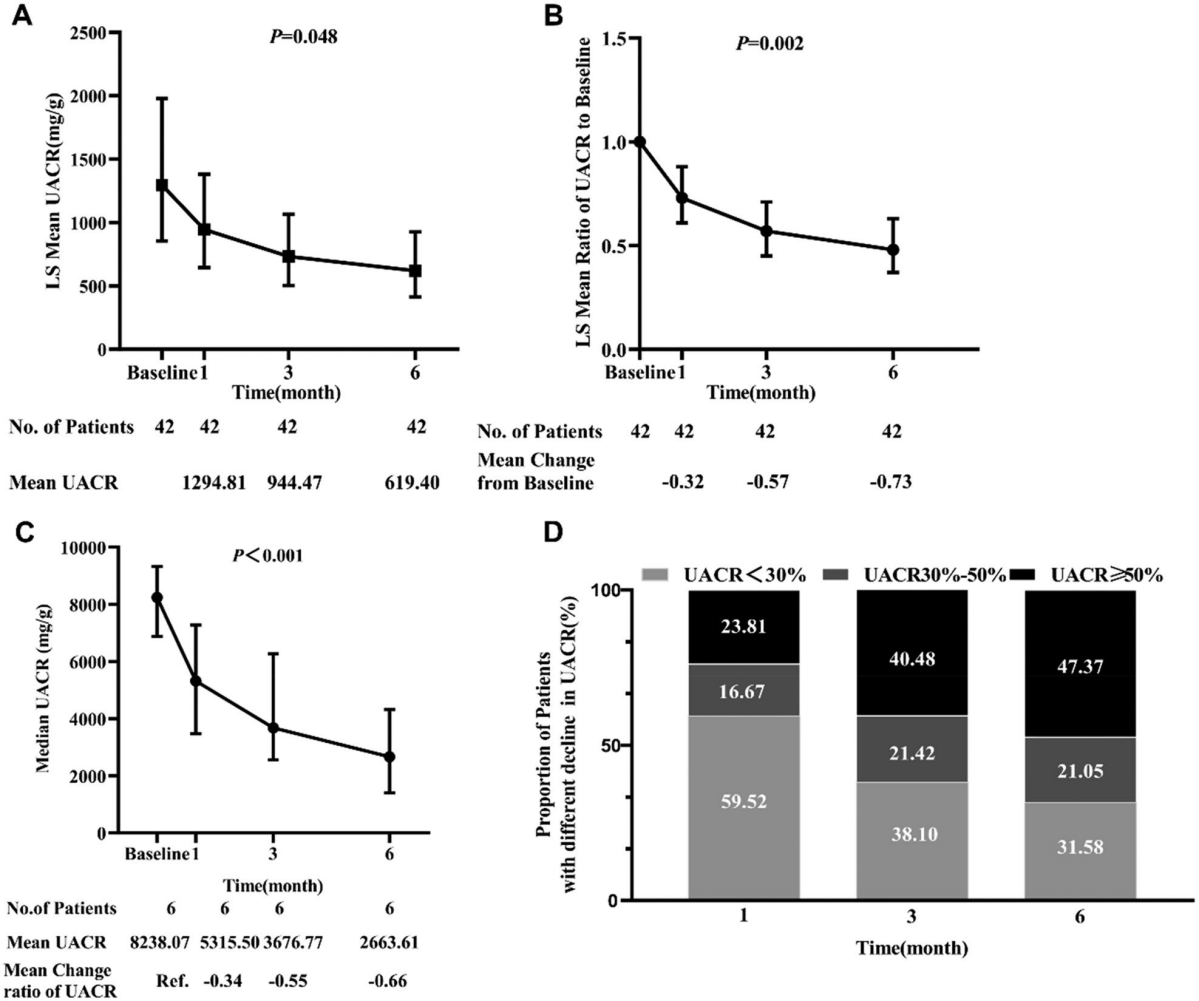
Finerenone effect on UACR (A), mean change of UACR from baseline (B), UACR of >5000 mg/g (C) and the proportion of patients with different declines in UACR (D) over time. Data are the least geometric mean and 95% confidence interval (CI) presented on a logarithmic scale. UACR: urine albumin-to-creatinine ratio.

Figure 2(A) shows that finerenone lowers the UACR sustainably throughout the 6-month study period. The changes in UACR least squares mean from baseline are shown in Figure 2(B). Finerenone reduced UACR by 32% at month 1 and 73% at month 6. On further subgroup analysis, UACR decreased by 66% in the subgroup with UACR of greater than 5000 mg/g (six patients) at month 6 (Figure 2(C)). Moreover, a 30% or greater reduction of UACR increased over time as shown in Figure 2(D). The proportion of patients (30–50% reduction of UACR) increased from 16.67% at month 1 to 21.05% at month 6, and the proportion of patients (>50% reduction of UACR) increased from 23.81% at month 1 to 47.37% at month 6.

### Safety outcomes and adverse events

**Figure 3.**
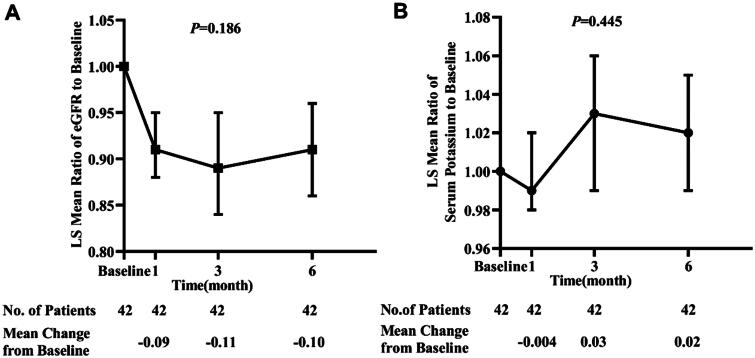
Finerenone effect on eGFR (A) and serum potassium (B) over time. Data are least squares (LS)-mean and 95% confidence interval (CI) presented on a logarithmic scale. eGFR: estimated glomerular filtration rate.

Figure 3(A) shows the eGFR slope over time by the finerenone effect. Compared with the baseline value, finerenone reduced eGFR by 9% at month 1. A smaller decline in eGFR from month 1 to the end of the study was seen. One patient who had stage 4 CKD discontinued finerenone due to acute kidney injury with a twofold increase in serum creatinine from baseline. However, this patient was prescribed SGLT2i and finerenone (20 mg) simultaneously. When SGLT2i and finerenone were discontinued, his kidney function recovered.

During the 6-month observational period, a numerical increase in serum potassium concentration was observed at month 3 and 6 (Figure 3(B)). The incidences of hyperkalemia (>5.5 mmol/L) at months 1, 3, and 6 were 4.8%, 7.1%, and 4.8%, respectively. One patient developed hyperkalemia (>6.0 mmol/L), but his serum potassium returned to normal range eventually.

Overall, AEs were reported in 10 patients (23.8% of the patients) in this current study. One patient developed manageable itching and skin rash. None of the patients reported hypotension, breast pain, or gynecomastia in this current study (Table 2).

**Table 2.** Adverse events and serious adverse events in patients treated with finerenone.

Events	*n* (%) of participants
Any adverse events	10 (23.8)
Any serious adverse events	2 (4.7)
Any drug-related serious adverse events	2 (4.7)
Discontinuation of study medication due to adverse events	2 (4.7)
Discontinuation of study medication due to serious adverse events	2 (4.7)
Discontinuation of study medication due to serum potassium of ≥5.6 mmol/L but <6.0 mmol/L	0
Discontinuation of study medication due to serum potassium of ≥6.0 mmol/L	1 (2.4)
Glomerular filtration rate decreased	1 (2.4)
Skin rash	1 (2.4)

## Discussion

Recent clinical trials provided robust evidence that finerenone significantly improved kidney and cardiovascular AEs in patients with type 2 diabetes and CKD [8,9]. However, the benefits and risks of finerenone treatment in real-world settings need to be evaluated. Some prospective observational studies are ongoing to evaluate the effectiveness and safety of finerenone in the treatment of DKD in real-world practices [14].

As shown in the Chinese subanalysis study, the mean UACR was also higher in our study [15]. This may be due to the high-salt diet in northern China compared to other parts of the country. In this present study, the addition of finerenone to standard therapies alleviated proteinuria with a manageable safety profile. To the best of our knowledge, this was the first real-world study that investigated the renal effect of finerenone in DKD patients with the inclusion of DKD patients with severely elevated albuminuria (>5000 mg/g). Furthermore, the proportion of patients with severely elevated mean albuminuria levels (>3000 mg/g) in this current study was higher than that in the FIDELIO-DKD study (26.3% vs. 7.9%, respectively) [16]. Retrospective and *post hoc* analyses showed that early intervention in reducing albuminuria (>30%) was associated with the reduced risk of CKD progression or CV events [17,18]. At the end of this current study, more than half of patients (68.43%) had a 30% or greater reduction in UACR. This reduction might have been due to different pharmacological mechanisms. RASi, SGLT2i, and finerenone all have a small adverse effect on glomerular hemodynamics by suppressing intraglomerular pressure. These pharmacological properties might slow down CKD progression and lower proteinuria, resulting in long-term kidney protection. Recent studies have shown that SGLT2i in combination with finerenone can reduce albuminuria to a greater extent [16,19,20].

Furthermore, the direct anti-inflammatory and anti-fibrotic properties of finerenone by the inhibition of MR overactivation might have conferred a better effect in lowering proteinuria in this current study. Considering that administration of the above three medications can reduce intraglomerular pressure, this current study did not observe a greater renal function deterioration although a higher proportion of RAS inhibitors, SGLT2 inhibitors, and finerenone combination was used (Supplementary Figure 1). With a comparable effect of finerenone on eGFR in other studies, eGFR in our study decreased by 9%, 11%, and 10% at 1, 3, and 6 months, respectively. This may be due to the intraglomerular pressure reduction in combination therapy [8,21–23]. However, kidney function began to stabilize from the third month. Despite this hemodynamic change, vigilance is still warranted to prevent worsening renal function in clinical practice, especially when medications that cause a decrease in intraglomerular pressure are used concomitantly or when hypotension or hypovolemia is present.

At present, SGLT2 inhibitors or finerenone in addition to RAS inhibitors are recommended as the standard treatment for type 2 diabetes with CKD [11,12]. However, dual inhibition of the renin–angiotensin–aldosterone system instantly can increase the risk of acute kidney injury and hyperkalemia, and therefore, caution is warranted. On the contrary, SGLT2 inhibitors reduced hyperkalemia by enhancing distal tubular sodium delivery [19,24]. In animal model studies and *post hoc* analyses of randomized control trials, co-administration of SGLT2i and finerenone displayed additional cardiorenal benefits [19,25–28]. However, evidence from clinical trials and real-world settings is limited. Our clinical data first showed that 6 months of combination finerenone and SGLT2 inhibitors therapy in addition to RAS inhibitors resulted in greater reductions of UACR (73% reduction) (Figure 2(B)). Compared with the phase 2 clinical trial of finerenone [15], the patients in our study had higher UACR levels (1426.11 mg/g) and a higher proportion of patients used the combination of finerenone and SGLT2 inhibitors (90.5%). The finerenone and SGLT2 inhibitors regimen not only resulted in effectiveness but also was safe with a lower incidence of hyperkalemia (Supplementary Figure 3) [29]. The risk of hyperkalemia by adding finerenone to RAS inhibitors might have been mitigated by the potassium-lowering effect of SGLT2 inhibitors (Supplementary Tables 2–4).

Our study also had several limitations. First, this was a single-center retrospective study on the Chinese population. The extrapolation to other ethics groups should be cautioned. Second, the current study lacked a larger sample size, long-term observation, and randomization. Therefore, randomized control trials with larger sample size and long follow-up visits are needed to evaluate the co-administration of finerenone and SGLT2 inhibitors in diabetic or non-DKDs.

## Conclusions

Our study confirms the real-world benefits of finerenone treatment on UACR in the Chinese population with DKD. To date, this was the first real-world study that included DKD patients with severe UACR (>5000 mg/g) who had been excluded from the previous randomized clinical trials with finerenone. Furthermore, a triple regimen of RAS inhibitors, SGLT2i, and finerenone achieved a promising effectiveness in lowering UACR in this current study. Finally, the individualized effect of concomitant medications needs to be carefully evaluated in routine clinical practice, particularly when it is used in moderate to advanced CKD.

## Supplementary Material

supplementary materials.docx

## Data Availability

The data in this study are available from the corresponding author upon a reasonable request.
