# Supplementary material for: Effectiveness and safety of finerenone in diabetic kidney disease patients: a real-world observational study from China
Source: Ren Fail. 2024 Sep 9;46(2):2400541. doi: 10.1080/0886022X.2024.2400541 (PMC11385639; doi:10.1080/0886022X.2024.2400541)
Supplement: supplementary materials.docx [file IRNF_A_2400541_SM5165.docx]

Supplementary materials

Effectiveness and Safety of Finerenone in Diabetic Kidney Disease Patients: A Real-World Observational Study from China


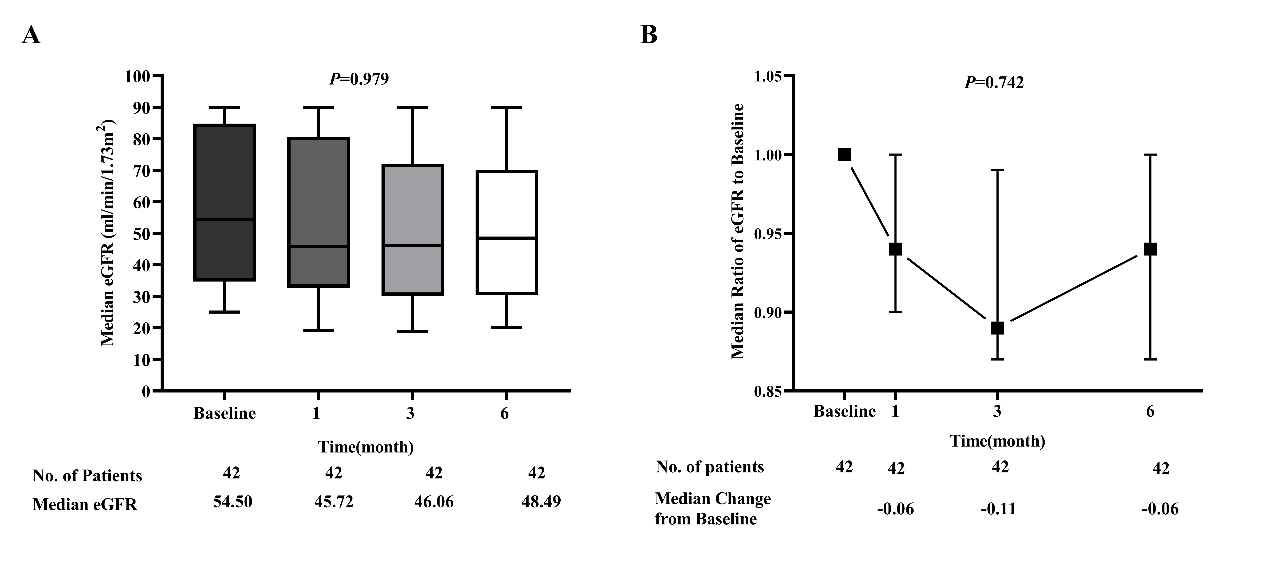


Supplementary Figure 1. The effect of finerenone on eGFR (A), median eGFR to the baseline value (B) over time. Data are given as median ± 95% confidence interval; eGFR, estimated glomerular filtration ratio.


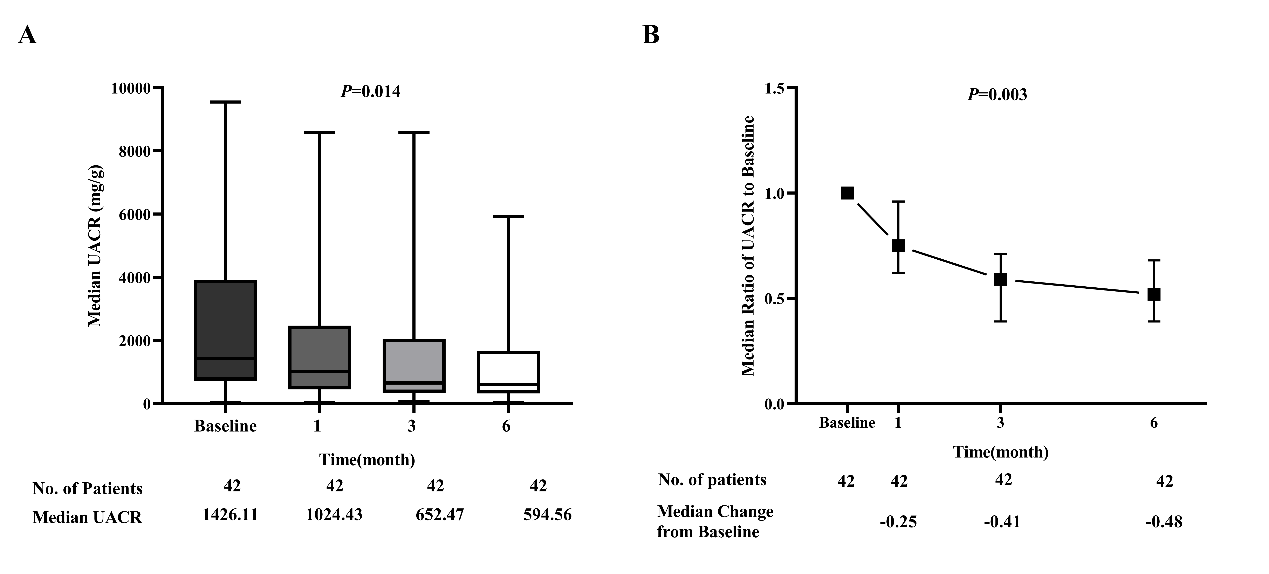


Supplementary Figure 2. The effect of finerenone on UACR (A), median UACR to the baseline value (B) over time. Data are given as median ± 95% confidence interval; UACR, urine albumin to creatinine ratio.


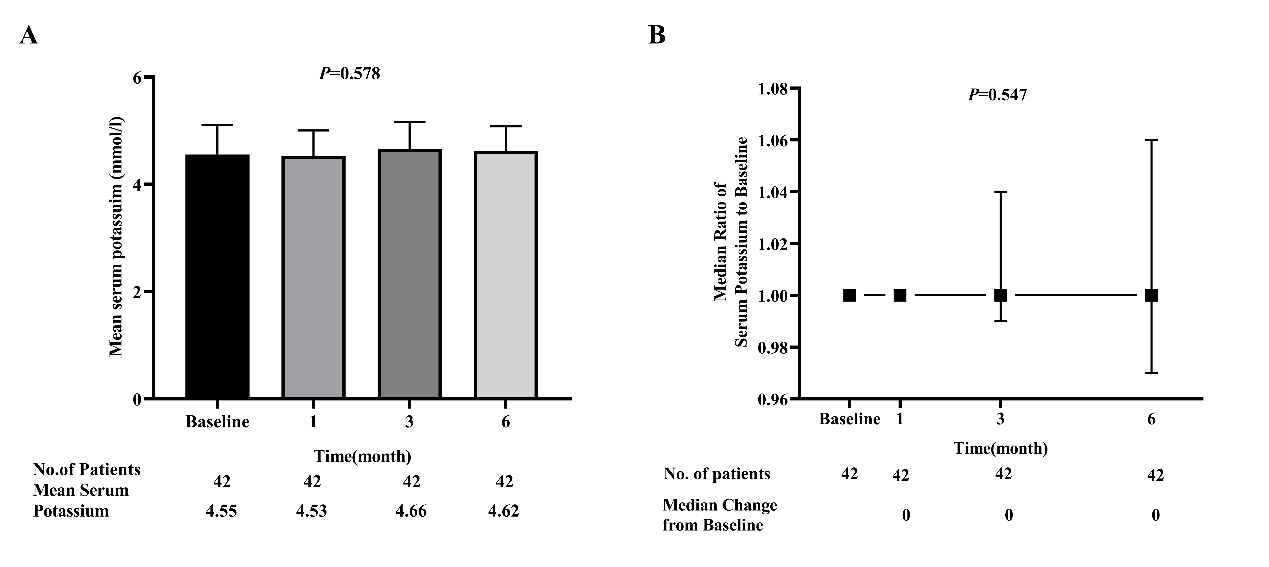
Supplementary Figure 3. The effect of finerenone on mean serum potassium level (A), and median ratio of serum potassium from baseline level (B) over time. Data in part A aregiven as mean ± 95% confidence interval. Data in part B aregiven as median ± 95% confidence interval.

Supplementary Table 1. Summary of ANCOVA for UACR, eGFR, and serum potassium level over time

| Variables | Baseline(n=42) | 1 mo(n=42) | 3 mo(n=42) | 6 mo(n=42) | P-value |
| --- | --- | --- | --- | --- | --- |
| UACR,Median (IQR), mg/g | 1426.11(755.42-3638.23) | 1024.42(480.07-2304.34) | 652.47(386.18-1853.39) | 594.56(343.50-1635.91) | 0.028 |
| eGFR,Median (IQR),mL/min/1.73m^2^ | 54.50(35.04-83.73) | 45.72(33.98-77.81) | 46.06(31.08-67.06) | 48.49(31.19-68.91) | 0.669 |
| Serum potassium, Mean ± SD, mmol/L | 4.55 ± 0.55 | 4.53 ± 0.48 | 4.66 ± 0.50 | 4.62 ± 0.46 | 0.578 |

UACR, urine albumin to creatinine ratio; eGFR, estimated glomerular filtration ratio; IQR, interquartile range; SD, standard deviation.

Supplementary Table 2. Summary of ANCOVA for change of UACR, eGFR, and serum potassium level from baseline values over time

| Variables | 1 mo/Baseline(n = 42) | 3 mo/Baseline(n = 42) | 6 mo/Baseline(n = 42) | P-value |
| --- | --- | --- | --- | --- |
| UACR, M (IQR), mg/g | 0.76 (0.50-1.00) | 0.59 (0.34-0.84) | 0.52 (0.31-0.75) | 0.033 |
| eGFR, M (IQR), mL/min/1.73m^2^ | 0.94 (0.87-1.00) | 0.89 (0.82-1.00) | 0.94 (0.86-1.00) | 0.742 |
| Potassium, M (IQR), mmol/l | 1.00 (1.00-1.00) | 1.00 (0.97-1.07) | 1.00 (0.94-1.07) | 0.547 |

UACR, urine albumin to creatinine ratio; eGFR, estimated glomerular filtration ratio; IQR, interquartile range.

Supplementary Table 3. Geometric least-squares mean change of UACR, eGFR, and serum potassium level from baseline

| Variables | Geometric least-squares mean change (95% CI) | Geometric least-squares mean change ratio(95% CI) | P-value |
| --- | --- | --- | --- |
| UACR ratio |  |  | 0.002 |
| 1 mo/Baseline | 0.73(0.61-0.88) | -0.32(-0.76-0.13) | 0.126 |
| 3 mo/Baseline | 0.57(0.45-0.72) | -0.57(-1.03-0.11) | 0.006 |
| 6 mo/Baseline | 0.48(0.37-0.61) | -0.73(-1.22-0.24) | ＜0.001 |
| eGFR ratio |  |  | 0.186 |
| 1 mo/Baseline | 0.91(0.87-0.94) | -0.09(-0.23-0.05) | 0.110 |
| 3 mo/Baseline | 0.89(0.83-0.95) | -0.11(-0.26-0.03) | 0.047 |
| 6 mo/Baseline | 0.91(0.85-0.96) | -0.10(-0.24-0.05) | 0.091 |
| Serum potassium ratio |  |  | 0.445 |
| 1 mo/Baseline | 1.00(0.18-1.01) | -0.004(-0.046-0.04) | 0.854 |
| 3 mo/Baseline | 1.03(1.00-1.06) | 0.03(-0.22-0.07) | 0.228 |
| 6 mo/Baseline | 1.01(1.00-1.03) | 0.02(-0.03-0.07) | 0.395 |

The UACR, eGFR, and serum potassium level are logarithmically normal, and data aretransformed. UACR, urine albumin to creatinine ratio; eGFR, estimated glomerular filtration ratio; CI, confidence interval.

Supplementary Table 4. Geometric least-squares means of UACR, eGFR, and serum potassium level

| Variables | Mean (95% CI) | Mean difference (95% CI) | P-value |
| --- | --- | --- | --- |
| UACR |  |  | 0.048 |
| Baseline | 1294.81(842.25-1899.75) |  |  |
| 1 mo | 944.47(647.41-1366.19) | -255.48(-545.71--195.03) | 0.256 |
| 3mo | 732.23(511.03-1049.84) | -318.15(-492.57--151.64) | 0.041 |
| 6 mo | 619.40(409.56-898.50) | -323.08(-447.89--107.47) | 0.009 |
| eGFR |  |  | 0.672 |
| Baseline | 51.56(44.67-58.49) |  |  |
| 1 mo | 47.06(40.77-53.74) | -4.10(-11.88-5.39) | 0.369 |
| 3 mo | 45.98(39.76-53.31) | -4.98(-12.40-4.09) | 0.259 |
| 6 mo | 51.89(45.09-59.20) | -4.79(-13.31-5.62) | 0.340 |
| Serum tassium |  |  | 0.578 |
| Baseline | 4.55(4.39-4.74) |  |  |
| 1 mo | 4.53(4.37-4.68) | 0.03(-0.18-0.25) | 0.789 |
| 3 mo | 4.66(4.52-4.81) | -0.11(-0.33-0.11) | 0.324 |
| 6 mo | 4.62(4.49-4.77) | -0.07(-0.30-0.14) | 0.531 |

The UACR and eGFR are logarithmically normal, and the data aretransformed.

UACR, urine albumin to creatinine ratio; eGFR, estimated glomerular filtration ratio; CI, confidence interval.
